# The Set2/Rpd3S Pathway Suppresses Cryptic Transcription without Regard to Gene Length or Transcription Frequency

**DOI:** 10.1371/journal.pone.0004886

**Published:** 2009-03-19

**Authors:** Colin R. Lickwar, Bhargavi Rao, Andrey A. Shabalin, Andrew B. Nobel, Brian D. Strahl, Jason D. Lieb

**Affiliations:** 1 Curriculum in Genetics and Molecular Biology, University of North Carolina at Chapel Hill, Chapel Hill, North Carolina, United States of America; 2 Department of Statistics and Operations Research, University of North Carolina at Chapel Hill, Chapel Hill, North Carolina, United States of America; 3 Department of Biochemistry and Biophysics, University of North Carolina School of Medicine, Chapel Hill, North Carolina, United States of America; 4 Department of Biology, Carolina Center for the Genome Sciences, and Lineberger Comprehensive Cancer Center, University of North Carolina at Chapel Hill, Chapel Hill, North Carolina, United States of America; Duke University, United States of America

## Abstract

In cells lacking the histone methyltransferase Set2, initiation of RNA polymerase II transcription occurs inappropriately within the protein-coding regions of genes, rather than being restricted to the proximal promoter. It was previously reported that this “cryptic” transcription occurs preferentially in long genes, and in genes that are infrequently transcribed. Here, we mapped the transcripts produced in an *S. cerevisiae* strain lacking Set2, and applied rigorous statistical methods to identify sites of cryptic transcription at high resolution. We find that suppression of cryptic transcription occurs independent of gene length or transcriptional frequency. Our conclusions differ with those reported previously because we obtained a higher-resolution dataset, we accounted for the fact that gene length and transcriptional frequency are not independent variables, and we accounted for several ascertainment biases that make cryptic transcription easier to detect in long, infrequently transcribed genes. These new results and conclusions have implications for many commonly used genomic analysis approaches, and for the evolution of high-fidelity RNA polymerase II transcriptional initiation in eukaryotes.

## Introduction

A primary function of eukaryotic genomes is to serve as the template for RNA transcripts that encode proteins. The exact location along the DNA where these transcripts initiate, and how frequently they are initiated, is highly regulated [1], [2], [3]. An important component of directing initiation events to the 5′ end of genes is regulating chromatin in such a way that a more open configuration is situated at the 5′ end, while DNA downstream of the promoter is less accessible to transcription factors [4], [5], [6]. If transcription-coupled chromatin remodeling is perturbed, as occurs in strains harboring mutations in the genes encoding Spt6, Spt16, and components of the Set2/Rpd3S pathway, inappropriate transcriptional initiation is found to occur at places within protein-coding regions [7], [8], [9], [10], [11], [12]. Therefore, the precise organization of chromatin along transcription units is critical to directing transcription factors and RNA polymerase II (RNA Pol II) to appropriate start sites within genes [3], [13], [14], [15].

The Set2 (Kmt3) enzyme methylates H3K36 in RNA Pol II transcribed portions of the genome, and is targeted to genes through its association with the phosphorylated C-Terminal Domain of the elongating RNA polymerase [16], [17], [18], [19], [20], [21]. Methylated H3K36 residues are then recognized by the Eaf3 subunit of the Rpd3S complex, leading to deacetylaytion of local histones by Rpd3 [9], [11]. This deacetylation is hypothesized to be important for maintaining the integrity of chromatin following transcription, thereby inhibiting the assembly of transcription factors at inappropriate or “cryptic” sites within genes [7], [9], [10], [11], [12], [19], [22]. When Set2 or other components of this process are disabled, inappropriate transcripts arise from within coding regions. The origin and potential function of these cryptic initiation sites has been the subject of intense investigation. One foundational study concluded that “cryptic” transcription occurs preferentially in long genes and in genes that are infrequently transcribed [12]. These conclusions suggest that particular gene types are more dependent on the Set2/Rpd3S pathway for their function than others. If true, this conclusion would imply that this class of genes is under a special selective pressure to suppress cryptic transcription, with potentially wide-ranging implications for the evolution of eukaryotic genomes. Here, we performed new experiments to re-examine the findings of earlier studies. We conclude that the “cryptic” transcription normally suppressed by the Set2/Rpd3S pathway occurs throughout the genome and does not appear to be associated either positively or negatively with gene length or transcriptional frequency.

## Results and Discussion

### Systematic ascertainment bias in previous characterizations of cryptic transcription may have influenced data analysis

It has been reported that longer genes, and genes that are infrequently transcribed, are particularly dependent on a mechanism of chromatin-mediated protection against cryptic transcription [12]. To identify genes that relied on the Set2/Rpd3S pathway to suppress cryptic transcription, Li *et al.* directly compared RNA prepared from *set2Δ* and wild-type strains using DNA microarrays. The authors then predicted the occurrence of cryptic transcripts by comparing the ratio of probe values measured at the 5′ end of the gene to the ratio of probe values at the 3′ end of the gene. A higher ratio at the 3′ end indicated an aberrant transcript that initiated somewhere downstream of the natural 5′ promoter. Based on the set of genes identified in this manner, it was concluded that cryptic transcription occurs preferentially in long genes, and in genes that are infrequently transcribed.

However, the previous experiments and analyses did not account for several important factors in collecting and analyzing the data. First, if cryptic transcription occurs at random along the genome, then it is more likely to occur in longer genes. Second, by the same reasoning, longer genes are more likely to contain multiple cryptic initiation sites. This would make microarray detection of cryptic initiation easier for longer genes, especially when the number of probes per gene is low, because multiple initiation events may be detected and interpreted as a single event. Third, a cryptic initiation event that is flanked on either side by a larger number of microarray probes, as occurs in longer genes, will be easier to detect because more measurements are taken on either side of the initiation event. Finally, cryptic transcription is inherently easier to detect on genes that are transcribed at low levels [23]. Consider two equally-sized genes, both of which have a cryptic promoter in the same position along the gene. In both cases, assume that cryptic promoter produces 5 mRNAs per hour. However, one of the genes is transcribed from its natural promoter at 1 mRNA per hour, and the other is transcribed at 50 mRNAs per hour. Assuming equal mRNA stabilities, the ratio produced at the 3′ end by the cryptic transcript of the highly expressed gene will be 55/50 = 1.1, while the ratio at the 3′ end of the infrequently transcribed gene will be 6/1 = 6. Therefore cryptic transcription that occurs in frequently transcribed genes (ratio 1.1) is much more difficult to detect than a cryptic promoter of equal strength from an infrequently transcribed gene (ratio 6). This would be especially true if the level of transcription at a given cryptic promoter is generally independent of the level of transcription from the natural promoter, as appears to be the case [9].

Given these potential biases, we were motivated to revisit the previous experiments with a higher-resolution detection platform, and to develop an analysis method that explicitly accounted for the confounding factors described above.

### At the level of raw data, new higher-resolution maps of Set2-dependent cryptic initiation are consistent with previous maps

As in the previous study, we prepared RNA from *set2Δ* and wild-type strains, and labeled them for direct comparison through microarray hybridization (**Methods**). In our case, the samples were applied to DNA microarrays containing 385,000 probes, which corresponds to an average start-to-start probe spacing of 31 bp, and an average of 51 probes per open reading frame (ORF). In the previous experiments, the arrays contained 40,174 probes, which corresponds to an average start-to-start spacing of 261 bp and an average of 4.6 probes per ORF. The higher resolution in the new study is critical: in the previous study 676 ORFs contained only a single probe, meaning that it was impossible to detect cryptic transcription in those genes, and 983 contained only two probes, meaning a call would be dependent on the value from a single probe. The higher resolution of our data afforded us the opportunity to call multiple cryptic initiation events within a single gene, rather than being limited to identifying genes in which one or more initiation events may have occurred. We therefore employed a statistically principled change-point detection algorithm to identify the step-like transitions in the log intensity ratios across a transcription unit [24]. These “transitions” represent putative cryptic initiation events.

The algorithm operates on a gene-by-gene basis, and in a sequential fashion to detect the existence and location of potential cryptic initiation events. At each probe within a gene, it compares the measured values (Z-scores) to the left and the right of the probe using a standard F-test. If the maximum F-statistic along the gene exceeds a pre-defined significance threshold (for the given gene length), the location at which the maximum is achieved is identified as a transition point. The search procedure is then recursively applied to the observations lying to the left and to the right of the transition point. Further details of the algorithm and the scaled *supF* statistic are presented in **Methods**.

**Figure 1:**
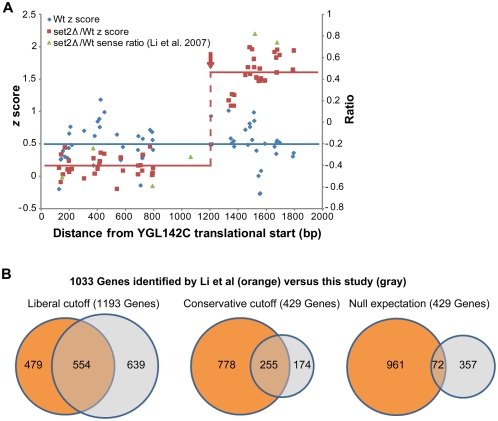
Detection of cryptic initiation sites in *S. cerevisiae* strains lacking Set2. (A) YGL142C exhibits cryptic initiation in the set2*Δ* strain (red, *supF* 29.7), but not wild-type (blue). Plotted along the length of YGL142C are *z* scores of wild type (Wt) RNA raw probe intensity values (blue); *set2Δ* RNA/Wt RNA (red) and *set2Δ*/Wt ratios from Li et al. 2007 (green). The transition probe detected by our algorithm is marked by a dashed red line and red arrow. The solid lines represent the average *z* score for Wt (blue) and *set2* *Δ*/Wt values (red) before and after the cryptic initiation event. This cryptic initiation event was confirmed by Northern blotting (not shown). (B) Genes identified as containing cryptic initiation sites according to our liberal (*supF* 3.0, 1193 genes) or conservative (*supF* 9.0, 429 genes) criteria were compared to the genes identified as containing a cryptic initiation event from Li et al. 2007 (1033 genes, orange). Shown at far right is the average intersection of 429 genes chosen at random with the Li et al set.

We selected two significance thresholds which led to family-wise error rates of roughly 10^−10^ and 10^−26^, and identified 1193 and 429 genes, respectively, with at least one cryptic initiation event (**Table S1**). Individual genes identified by the algorithm as containing cryptic initiation events typically exhibit internal transitions that are clear by visual inspection (**Figure 1A**). Furthermore, genes characterized previously as containing cryptic transcripts in *set2Δ* strains, including *FLO8* (*supF* = 3.62) *STE11* (*supF* = 9.02) and *PCA1* (*supF* = 70.9) were identified by our algorithm. Using the more stringent cutoff, 59% of the genes we identified were identified previously as containing a cryptic initiation event [12]. We find this concordance striking, especially because the earlier study made use of a different microarray platform, a different RNA labeling method, and a different method of identifying cryptic initiation sites (**Figure 1B**). Indeed, examination of individual loci reveals that the raw data from this study and the previous study are consistent with each other (**Figure 1A**). The concordance of our raw data with the raw data from the lower-resolution study, along with the utility of our data in the identification of genes previously characterized as containing cryptic transcripts, provides support for our experimental design, analysis methods, and threshold selections.

### More cryptic initiation sites are detected in long genes, and these cannot be accounted for solely by correcting for gene length

**Figure 2:**
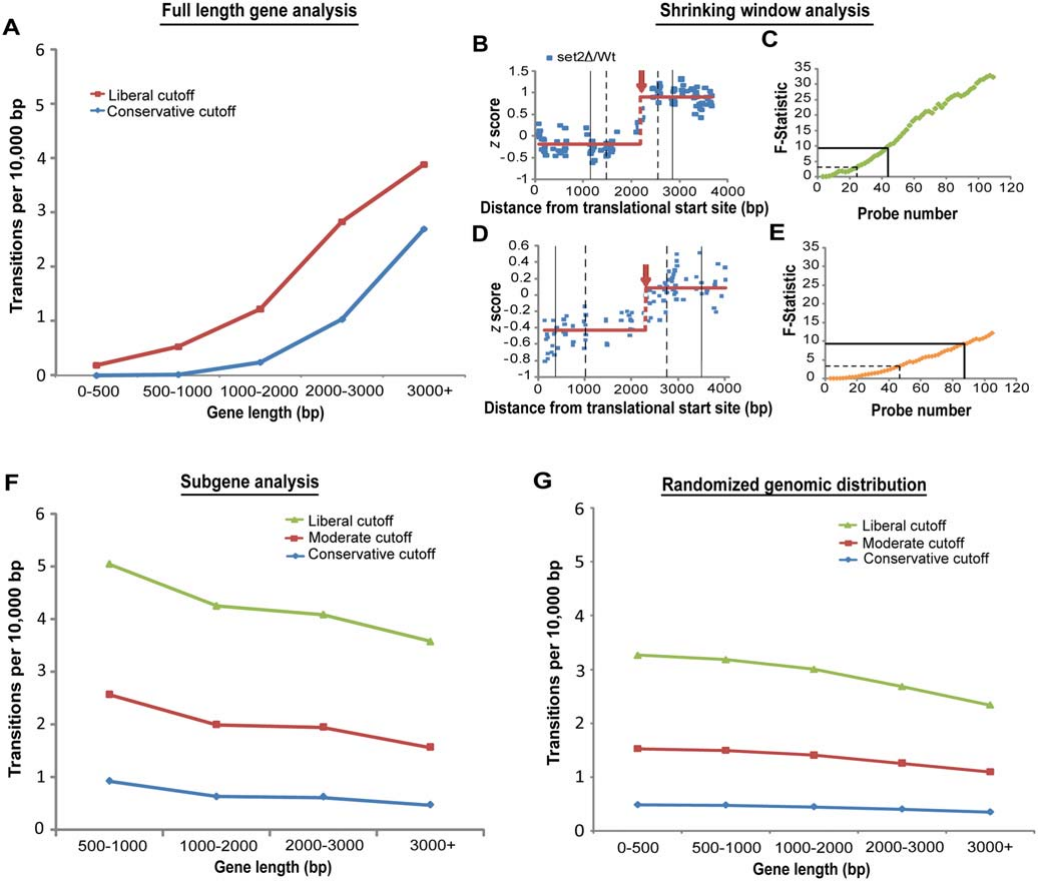
Cryptic initiation events occur without regard to gene length. (A) The number of cryptic initiation events detected per base increases with gene length. Shown is the detection rate for five gene-length bins at conservative (*supF* 9, 801 transitions, blue) and liberal (*supF* 3, 1757 transitions, red) cutoffs. (B–C) Reducing the number of probes flanking a transition event causes statistical significance to decrease. Plotted along the length of YDR104C are *z* scores of *set2Δ* RNA/Wt RNA. The solid horizontal lines represent the average *z* score for the *set2* *Δ*/Wt values (red) before and after the cryptic initiation event. The black solid and dashed vertical lines correspond to the number of probes required to achieve the *supF* 9 and *supF* 3 cutoffs respectively. Panel C plots the calculated *supF* values (y-axis) as a window centered on the detected transition shrinks, causing fewer probes to be available for transition detection (x-axis). The solid and dashed black lines call attention to the *supF* 9 and *supF* 3 cutoffs. (D–E) Same as B and C, but for YAL026C. (F) Detection of cryptic initiation sites using subgenes of uniform length eliminates the relationship between gene length and cryptic initiation frequency. Cryptic initiation rates for subgene analysis (Methods) using liberal (green; *supF* = 0.58, 2417 transitions), moderate (red; *supF* = 0.97, 1135 transitions), and conservative (blue; *supF* = 1.8, 364 transitions) cutoffs are plotted for different gene-length bins. (G) Random distribution of cryptic initiation events mimics the real distribution discovered after controlling ascertainment bias. Plot is the same as panel F, but transitions were randomly assigned to ORF probes (liberal; 2417 transitions, moderate; 1135 transitions, conservative; 364 transitions). Compare G and F. The slight downward trend in both plots may result from a slightly lower probe density in longer genes, which was not corrected for.

Previous studies have concluded that longer genes are especially prone to cryptic transcription upon deletion of *SET2* [12]. This conclusion was based on the observation that long genes were more likely to be identified as containing a cryptic promoter than short genes. To determine if this observation could be explained solely by the fact that longer genes afford more opportunity for a cryptic event to occur, we grouped all genes according to size. For each group, we then calculated the rate of cryptic initiation (measured in transitions per base pair, **Figure 2A**). Measuring the rate of transitions, rather than the absolute number of transitions, for genes binned by length is a simple way to correct for gene length. The positive correlation between gene length and the rate of transitions per base shows that transitions are indeed detected more often in the context of longer genes, and that the additional transitions detected in long genes cannot be accounted for simply by correcting for gene length.

### An ascertainment bias makes detection of cryptic transcripts easier in longer genes

Any method for change-point detection, in this case applied to cryptic transcription, will have greater power to identify a transition event that is flanked on either side by a large number of microarray probes. Thus in small genes, which are represented by fewer probes, transitions are more difficult to detect. We wondered if this ascertainment bias might explain the observed higher rate of cryptic transcript detection in longer genes. To investigate this, we took all of the cryptic initiation sites we detected, and computationally reduced the number of probes surrounding each transition. In each case, this was accomplished by examining a shrinking window centered on each transition point. Despite these transitions being called significant when the full gene length is used, when the shrinking window was applied, statistical confidence was reduced, often below our selected cutoffs, as the window size approached 500 bp (**Figure 2B–2E**).

We conclude that our method, and indeed any statistically-principled method for cryptic initiation analysis using discrete expression-based probes, can more readily identify sites of cryptic initiation in long genes, where more probes flank every putative site. As a result, a cryptic initiation site of a given magnitude is more likely to be detected in longer genes containing many probes than in shorter genes with relatively few probes.

### Controlling probe-number ascertainment bias eliminates the relationship between cryptic initiation events and gene length

If long genes truly contain more sites of cryptic initiation than short genes, those additional sites of initiation should still be detected when long and short genes are placed on equal footing with regard to detection. To test this, for each gene over 500 bp in length, we artificially broke that gene into as many non-redundant approximately 500 bp “subgenes” as possible (**Methods**). We then re-ran our detection algorithm on these newly created “subgenes”, which were of relatively uniform length. To see if longer genes really harbored more cryptic initiation events, we computationally stitched the genes back together, binned the genes according to their length, and again calculated the rate of cryptic initiations per base. This treatment of the data holds constant both the probe-number bias and the bias caused by multiple cryptic initiations in the same gene, both of which favor detection of cryptic initiation events in longer genes. We found that after this treatment, long genes no longer harbor more cryptic initiation events. Indeed, they may harbor fewer cryptic initiation events per unit length (compare **Figures 2A and 2F**). Therefore, controlling ascertainment biases inherent in transcript-based detection by microarray eliminated the relationship between the occurrence of cryptic initiation events and gene length.

### Sites of cryptic initiation are distributed randomly with regard to gene length

Our analysis of cryptic initiation events is consistent with such events being distributed throughout transcription units without respect to their length. To test this hypothesis, we assigned each detected transition at random to an ORF probe, and compared several features of the randomly assigned transitions to these same features under the observed (not randomized) transition locations. As was the case with the observed locations, and as expected, when transitions were randomly distributed, longer genes contained more transitions. However, when the transition rate *per base* was calculated using the random assignments, there is no relationship between transition rate and length, which is nearly identical to what we observe with real data when the cryptic initiation calls are made using uniform-length subgenes, which control probe-number ascertainment bias (**Figure 2G**; compare to **Figure 2F**). The fact that randomly assigned transitions, which do not depend on a detection algorithm, so closely mimic the observations made with real data when length biases are controlled, strongly suggests that real cryptic initiation events are distributed randomly in relation to gene length.

### Sites of cryptic initiation are distributed randomly with regard to transcription rate

**Figure 3:**
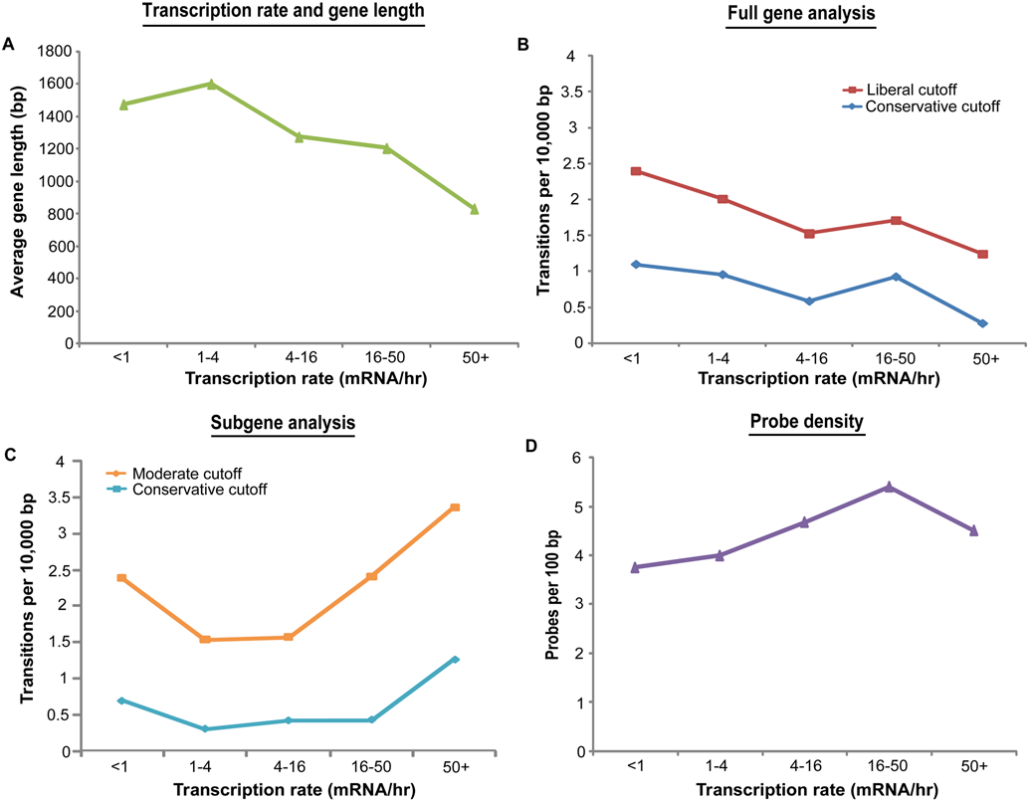
Cryptic initiation events occur without regard to transcriptional frequency. (A) Infrequently transcribed genes tend to be long. The average gene length for each transcription-rate bin is shown. (B) Without correcting for gene length, infrequently transcribed genes appear to have a higher rate of cryptic initiation. Genes were binned according to their transcription rate, and the cryptic initiation rate was determined for each bin. (C) Detection of cryptic initiation sites using subgenes of uniform length eliminates the inverse relationship between gene transcription frequency and cryptic initiation frequency. There may in fact be a trend toward more cryptic initiation in heavily transcribed genes. (D) Probe density cannot account for the observed relationship between transcription frequency and cryptic initiation frequency. Probe coverage is fairly uniform across gene transcription rate bins.

Previous studies reported that infrequently transcribed genes are especially prone to cryptic initiation events in the absence of Set2 [12]. However, as has been noted by others, gene length and transcription rate are not independent variables [18], [25]. In particular, long genes tend to be transcribed less frequently (**Figure 3A**). We sought to determine if any relationship between transcription rate and cryptic initiation rate remained after controlling for ascertainment biases due to gene length. First, we binned genes according to their transcription rate [25], and plotted the number of cryptic initiations per base for each of the bins according to our full-length gene analysis. Consistent with the previous reports [12], we observed an apparent inverse relationship between cryptic initiation rate and transcription rate (**Figure 3B**). However, we noted that the plot looked very similar to the relationship between gene length and transcription rate (**Figure 3A**). Next, we performed the same analysis, but this time using our subgene analysis. As described above, the subgene analysis controls the probe-number bias that favors detection in long genes. With this bias controlled, we now find that more heavily transcribed genes are, if anything, more likely to contain cryptic initiation sites (**Figure 3C**), in contradiction to the previously reported results. Furthermore, this increased rate of cryptic initiation among highly transcribed genes cannot be accounted for by differences in probe coverage among this class of genes (**Figure 3D**).

### The ascertainment biases reported here are likely widespread

Although not addressed directly by our manuscript, the same biases that cause long and infrequently transcribed genes to appear to be more susceptible to perturbations of the Set2/Rpd3S pathway could also make it appear as if such genes are differentially associated with histone modifications or other genomic features. These biases are exaggerated by the popular “modified average gene analysis” method [12], [26], [27], [28], which unnaturally forces every gene into a unit length, despite variations in real gene length of over an order of magnitude. Not only are such representations of dubious biological relevance, they are also suspect from a statistical standpoint because the number of data points underlying the “modified average gene” vary dramatically depending on real gene length. We are optimistic that technologies such as next-generation DNA sequencing will partially overcome the ascertainment biases we report here, but when transcripts are analyzed, even these approaches will be susceptible to such effects since longer and more frequently transcribed genes will accumulate more sequence reads.

### Conclusions

The hypothesis that infrequently transcribed long genes are particularly dependant on the Set2/Rpd3S pathway for accurate transcription is not supported by our data. Instead, our analysis indicates that Set2 suppresses cryptic transcription in wild-type cells across the genome in a manner that is, to first order, independent of gene length and gene frequency. This is a significant conclusion because it is consistent with previous reports that H3K36 methylation levels in yeast are largely independent of transcription rate [18], and because it obviates the need to posit a biological or evolutionary mechanism that accounts for a link between suppression of cryptic transcription and gene length or transcriptional frequency. It appears more likely that cryptic transcripts arise due to chance occurrences of DNA sequences that have the capacity to inappropriately recruit transcription initiation factors, or initiate transcription, for example sequences resembling a TATA box [7] or nucleosome-excluding elements [29], [30], [31]. Alternatively, sites of cryptic transcription could be distributed arbitrarily relative to length and transcription rate, but still represent functional internal promoters that are conditionally active [23]. While to date there is no evidence of a function for cryptic transcripts in yeast, the use of alternative start sites is extremely widespread in eukaryotic genomes, and it is likely that the Set2/Rpd3S pathway influences the evolution of alternative promoters in more complex eukaryotes.

## Materials and Methods

### RNA preparation

Wild type (BY4741) and *set2Δ* (BY4741) strains were grown at 30°C in YPD (1% yeast extract, 2% peptone, 2% dextrose) to an OD_600_ of 0.6–0.8. For each of the three replicates, total RNA was extracted by acid-phenol method [16]. Lack of RNA degradation was confirmed by gel electrophoresis. Double-stranded cDNA was prepared using an Invitrogen SuperScript™ (Cat No. 11917-010) primed with Oligo(dt) and random hexamers. For each replicate, the wt and *set2Δ* cDNA were independently fluorescently labeled and comparatively hybridized to high-resolution 385K *Saccharomyces cerevisiae* CGH arrays (2005-08-16_SCER_WG_CGH) with Tm-normalized probes. In one of the replicates, assignment of the fluorescent label was reversed. *Z* scores were calculated for each replicate based on the log_2_ (635/532 intensity) for each probe. The average *z* score value for each probe among the three replicates was used for further analysis. For Figure 1A, wild-type values were calculated by median centering the raw wild-type probe values from each array. Z scores were then calculated for each array, and the average z score for each probe across the three replicates was used for subsequent analysis. Raw microarray data is available via GEO accession number GSE13310.

### Cryptic initiation analysis

To detect cryptic initiation events, we employed a change-point detection algorithm [24]. The algorithm is applied independently to each gene and operates in a sequential fashion. At each probe within a gene, the algorithm compares the average measured probe values (*z* scores) to the left and to the right of the probe using a standard F-statistic. We then calculate the maximum F statistic appearing along the gene, which is known as the *supF* statistic [32], and compare a scaled version of this statistic to one of two pre-defined significance thresholds. If the scaled *supF* statistic exceeds a significance threshold, the corresponding location of the observed maximum F-statistic is identified as a cryptic initiation point. The search procedure is then applied separately and independently to the observations lying to the left and to the right of the detected cryptic initiation point. This process of splitting and searching for potential cryptic initiations continues until no significant transition (as judged by the scaled *supF* statistic) can be found in a given interval.

The number of probes varies greatly across genes, ranging from few to several hundreds. The moderate number of probes for most genes does not allow us to use the asymptotic distribution of the *supF* test statistic. For each gene length *n_g_*, we estimated the distribution of the *supF* statistic under the null model of no transition points by calculating the *supF* statistic for 100,000 samples of *n_g_* independent standard normal random variables. In many cases the observed *supF* statistic was substantially larger than all 100,000 simulated values. To be able to directly compare the observed *supF* statistics, we divide them by the corresponding 0.1% quantile of the simulated distribution. The resulting scaled *supF* statistics have more similar distributions across different gene lengths n*_g_* under the null model. To achieve higher power and consistent asymptotic properties of the test, we required the cryptic initiation points to be at least α = 5% of the gene probes apart of each other and the ends of the gene.

Although the distribution of the scaled *supF* statistic is unknown, we are able to obtain an upper bound for the probability that *supF* is larger than a given number A. The scaled *supF* is larger than *A* if 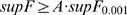, where 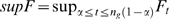 by definition, *supF_0.001_* is the 0.1% quantile of the *supF* statistic obtained from simulations and 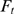 is the standard F-statistic for potential transition at probe *t*. Using Bonferroni correction we get the upper bound:
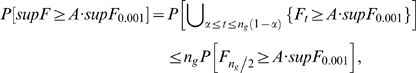
where the tail probability of the standard F-statistic (under the null model) in the right hand side term can be calculated precisely.

We considered scaled *supF* thresholds of 3 and 9. Using the approach described above and applying the Bonferroni correction for the number of genes we found that these thresholds correspond to nominal family-wise error rates of less than 10^−10^ and 10^−26^ respectively. With these thresholds, we identified 1193 and 429 genes respectively with at least one cryptic initiation. Throughout the manuscript, transition rate (transitions per bp) was calculated by totaling the number of cryptic initiations that are calculated at defined cutoffs for a particular class of genes and then dividing this number by the total length of genes analyzed within that class.

### Shrinking window, subgene, and randomization analysis

Shrinking window analysis was performed using ORFs that had a single cryptic initiation site with a scaled supF statistic over 9. Windows were centered so that an equal and maximum number of probes existed on either side of the cryptic initiation, such that the entire window was contained within the ORF. Scaled supF Statistics were then recalculated iteratively as one probe on each end of the window was removed.

Subgene analysis was performed by breaking full-length ORFs over 500 bp in length into as many non-overlapping, approximately 500 bp subgenes (with deviations from 500 bp being the result of probe coverage). Subgenes were then analyzed individually using our detection algorithm. The called transitions for each subgene were then mapped back to the gene from which the subgene was derived. The resulting distribution of called cryptic initiation events was used for subsequent analysis, with the gene lengths in this case being defined by the total length of the constituent subgenes.

To randomly distribute cryptic initiation events, probes were chosen at random from all probes within ORFs. Each gene containing a chosen probe was then defined as having a transition. This was repeated 1000 times each for 364, 1135 and 2417 transitions respectively. Averages from the 1000 iterations were used for subsequent analysis.

## Supporting Information

Table S1 A table representing all detected transitions using a change point algorithm at two different cutoffs as described in the text.
(0.47 MB XLS)
